# The Association among Hypothalamic Subnits, Gonadotropic and Sex Hormone Plasmas Levels in Alzheimer’s Disease

**DOI:** 10.3390/brainsci14030276

**Published:** 2024-03-14

**Authors:** Edward Ofori, Anamaria Solis, Nahid Punjani, on behalf of the Alzheimer’s Disease Neuroimaging Initiative

**Affiliations:** 1 College of Health Solutions, Arizona State University, Phoenix, AZ 85004, USA; 2 Department of Social Work, University of Texas at El Paso, El Paso, TX 79968, USA; asolis35@miners.utep.edu; 3 College of Medicine and Sciences, Mayo Clinic, Phoenix, AZ 85054, USA

**Keywords:** Alzheimer’s disease, hypothalamic–pituitary–gonadal axis, neurodegeneration, cognitive status, gonadotropic hormones, sex hormones, hypothalamic volume, neuroendocrine dysfunction

## Abstract

This study investigates the sex-specific role of the Hypothalamic–Pituitary–Gonadal axis in Alzheimer’s disease progression, utilizing ADNI1 data for 493 individuals, analyzing plasma levels of gonadotropic and sex hormones, and examining neurodegeneration-related brain structures. We assessed plasma levels of follicle stimulating hormone (FSH), luteinizing hormone (LH), progesterone (P4), and testosterone (T), along with volumetric measures of the hippocampus, entorhinal cortex, and hypothalamic subunits, to explore their correlation with Alzheimer’s disease markers across different cognitive statuses and sexes. Significant cognitive status effects were observed for all volumetric measures, with a distinct sex-by-cognitive status interaction for hypothalamic volume, indicating a decrease in males but not in females across cognitive impairment stages. Regression analyses showed specific hypothalamic subunit volume related to hormone levels, accounting for up to approximately 40% of the variance (*p* < 0.05). The findings highlight sex differences in neurodegeneration and hormonal regulation, suggesting potential for personalized treatments and advancing the understanding of Alzheimer’s disease etiology.

## 1. Introduction

In addition to serving as a central regulatory system for the production and sex hormone modulation, the hypothalamic–pituitary–gonadal (HPG) axis exerts both organizational and activating effects across a variety of neural substrates [1]. Impairment of regulatory mechanisms of the HPG axis has been implicated in the pathophysiology of Alzheimer’s disease (AD), although the nature and extent of this relationship remain enigmatic [2]. The prevalence and pathology of AD exhibit marked sex differences, raising the question of whether alterations in the HPG axis have different effects on male and female brains, particularly hormone secretion [3].

The hypothalamus coordinates numerous physiological processes, including hormone secretion [4], circadian rhythms [5], and energy balance [6], that are disrupted in AD. As a result of its connection to hormones through the hypothalamic–pituitary–gonadal axis, neuroendocrine dysfunction may interact with the neurodegenerative cascade in AD. In response to the growing interest in brain-based imaging biomarkers for Alzheimer’s disease, it is imperative to examine the hypothalamus and its subregions further, as identifying volumetric changes may reveal new biomarkers to examine in conjunction with established markers for diagnosis and prognosis [7]. By closely monitoring hypothalamic structural integrity, researchers can gain a valuable insight into neuroendocrine disturbances and disease trajectory in patients with Alzheimer’s disease and potentially understand sex differences that occur within the disease.

Each of the hypothalamic subnuclei plays a distinct role in a variety of physiological processes, including the regulation of stress, the regulation of food intake, and the regulation of circadian rhythm [8]. The relationship between these subnuclei and circulating plasma hormonal markers could inform targeted therapeutic approaches. For instance, if the inferior tuberal region, involved in stress and metabolic regulation, is found to be associated with specific hormonal changes, interventions could be tailored to modulate this region or the associated hormonal pathways [2]. The negative feedback mechanisms involving circulating plasma levels of hormones are crucial for maintaining homeostasis. In the context of AD, dysregulated feedback could exacerbate neurodegenerative processes [3,7,9]. For instance, elevated levels of cortisol, a stress hormone, have been associated with increased amyloid-beta deposition, a hallmark of AD. In addition, studies have observed that aberrant levels of luteinizing hormone (LH) may contribute to the dysregulation of amyloid precursor protein processing, thereby exacerbating the amyloidogenic pathway [9]. Therefore, a well-functioning feedback system could serve as a protective mechanism, helping to maintain neural integrity and potentially slow down neurodegenerative processes.

Understanding the neuroendocrine factors contributing to AD symptoms may provide insights into why there are disparities in AD manifestation amongst women, downstream effects on cognition and potential interventions related to hormone function for prevention, risk aversion, or delayed progression. Previous work has indicated a potential link between sex hormones and cognitive decline, with LH also being found to be associated with plasma amyloid-beta in men in a small sample [10,11]. Recently, it was shown that the hypothalamus volume can distinguish between stages of AD [7]. However, there are no studies linking differences in hypothalamus volume with circulating hormones across sexes. We aimed to examine the assessment of hypothalamic volume compared to AD-relevant neurodegenerative (N+) magnetic resonance imaging (MRI) markers across cognitive normal (CN), mild cognitive impairment (MCI), and AD for males and females. We also examined the relationship between hypothalamic subunit volume and plasma LH, follicle stimulating hormone (FSH), progesterone (P4), and testosterone (T). We expect a distinct biological sex-by-cognitive status group relation for males and females for hypothalamic volume. There will be different subunit volume measures that will be related to hormone levels in males and females.

## 2. Methods and Materials

### 2.1. Participants

A cohort of 493 individuals from the Alzheimer’s Disease Neuroimaging Initiative 1 (ADNI1) was enlisted for the study. The cohort comprised individuals across a spectrum of cognitive functioning including CN, MCI, and Alzheimer’s Disease (AD). Demographic information including age, biological sex, and education alongside clinical assessments were collected. All participants underwent a comprehensive hormonal screening and a high-resolution T1-weighted magnetic resonance imaging (MRI) scan. All participants had assessments which included a Clinical Dementia Rating-Sum of Boxes (CDR-SB) [12,13], Mini-Mental State Exam (MMSE) [14], and Rey’s Auditory Verbal Learning Testing (RAVLT) at baseline [13].

The data that informed this article’s preparation came from the Alzheimer’s Disease Neuroimaging Initiative (ADNI) database, which can be accessed at adni.loni.usc.edu. Initiated in 2003 as a collaborative venture between the public and private sectors led by Principal Investigator Michael W. Weiner, MD, the ADNI aims to investigate whether a combination of serial MRI, PET, other biomarkers, and clinical plus neuropsychological assessments can serve to monitor the progression of mild cognitive impairment (MCI) and the early stages of Alzheimer’s disease (AD). For the latest details, see www.adni-info.org (15 May 2023). The criteria for ADNI eligibility and diagnostic classifications are described at http://www.adni-info.org/Scientists/ADNIGrant/ProtocolSummary.aspx (15 May 2023).

### 2.2. Cognitive Status Groups

Cognitively Normal (CN): Participants demonstrated normal memory function as evidenced by scores on the Logical Memory II subscale of the Wechsler Memory Scale—Revised, with specific cutoffs based on education level. These individuals must have a Mini-Mental State Exam (MMSE) score ranging from 24 to 30. A Clinical Dementia Rating (CDR) of 0 was required, indicating no significant impairment.

Mild Cognitive Impairment (MCI): Participants with mild cognitive impairment must also report memory complaints confirmed by a study partner. They were differentiated from cognitively normal individuals by abnormal memory function, falling below the education-adjusted cutoffs on the Logical Memory II subscale. Their MMSE scores were between 24 and 30. A Clinical Dementia Rating of 0.5 with a Memory Box score of at least 0.5 was also required.

Alzheimer’s Disease (AD): For individuals with Alzheimer’s disease, inclusion criteria included the presence of memory complaints, confirmed by an informant, and significantly impaired memory performance on the Logical Memory II subscale according to their educational attainment. Their MMSE scores should ranged between 20 and 26, with potential adjustments for educational level at the director’s discretion. A Clinical Dementia Rating of 0.5 or greater was indicative of their cognitive impairment.

### 2.3. Image Acquisition and Preprocessing

The MRI acquisition protocols were described in a previous report [15]. In brief, high-resolution T1-weighted magnetic resonance DICOM images were obtained using 1.5-Tesla MRI machines employing a sagittal three dimensional magnetization-prepared rapid gradient-echo sequence. This technique featured an approximate repetition time of 2400 ms, a minimum full echo time, an inversion time of 1000 ms, and a flip angle of 8°. It is important to note that scan parameters differed across sites, scanner models, and software versions.

### 2.4. Hypothalamic Segmentation

The automated segmentation tool in FreeSurfer 7.2 was employed to segment the hypothalamus and its subunits. The 5 subunits were (1) the anterior–superior hypothalamus; (2) the anterior–inferior hypothalamus; (3) the superior tuberal hypothalamus; (4) the inferior tuberal hypothalamus (4); and (5) the posterior hypothalamus [7]. This approach ensures precise identification and analysis of these critical brain regions. The tool leveraged a convolutional neural network to compute segmentation maps of the hypothalamus encompassing five distinct subregions. The segmentation maps were visually inspected to ensure accuracy and precision in delineation. Any misregistration of volumes was manually adjusted and reran through the segmentation tool.

### 2.5. Volumetric Analysis

Volumetric measures for the segmented hypothalamic subunits alongside entorhinal, hippocampal, and fusiform regions were computed with FreeSurfer 7.2. The final volumetric data used were derived by taking the actual volume of the selected region of interest and dividing by the intercranial volume to normalize across various head sizes.

### 2.6. Hormonal Metrics

Plasma levels of key gonadotropic hormones including follicle stimulating hormone (FSH), LH, and sex hormones testosterone (T) and progesterone (P4) were assayed using standardized hormonal assays [16]. The hormonal metrics were collated and readied for statistical analyses. We obtained plasma hormonal levels via the ADNI website based on recommendations from the Biomarkers Consortium Plasma Proteomics Project and extracted our sample using the ADNImerge package. To briefly go over the multi-tiered ADNI process: (1) blood samples were collected and interrogated in accordance with ADNI standard operating procedures detailed in the procedural manual (http://adni.loni.usc.edu/methods/ (15 May 2023)). (2) Plasma hormones were quantified in overnight fasting blood samples, obtained from participants before breakfast. A majority of blood samples were frozen under 120 min from collection; (3) whole-blood samples were collected into 10-mL BD lavender top K2EDTA-coated Vacutainer tubes and centrifuged within one hour after collection; (4) blood plasma was moved to a polypropylene transfer tube in dry ice then to the ADNI Biomarker Core Laboratory at the University of Pennsylvania; (5) 0.5 mL aliquots were prepared from plasma samples and stored in polypropylene aliquot tubes at −80 °C until they were analyzed.

Rules-Based Medicine, Inc. (Myriad RBM, located in Austin, TX, USA) conducted an analysis on plasma samples to measure the levels of 190 different analytes using a comprehensive multiplex immunoassay panel known as the Human Discovery Multi-Analyte Profile (MAP), which is based on the Luminex xMAP technology developed by RBM. This service, aimed at exploring the plasma samples using the human discovery map, is offered on a fee-for-service basis. Myriad RBM implements three tiers of quality control (QC) for each analyte, with the QC outcomes, the assays’ detection limits, and the dynamic ranges for each plasma analyte presented in the data primer. The variability between assays, or coefficients of variation (CVs), was calculated for each analyte across all plates. Any analyte that showed a CV greater than 25% in one or more QC checks was flagged, although this did not apply to the plasma cortisol levels. Comprehensive assay details and quantification techniques are outlined in the data primer available at the ADNI website, with additional in-depth documentation and validation reports accessible through Myriad RBM’s website and related reports [17]. The selection process targeted assays that accurately mirror the functioning of the hypothalamus.

### 2.7. Statistical Analysis

Demographic information was run with one-way ANOVA for the Age, Education, CDR-SB, MMSE, RAVLT, and estimated intracranial volume (eTIV). Chi-square analyses were run to determine any association between sex and cognitive status group. The significance was determined at the α = 0.05 level. Separate linear regression analyses were performed to ascertain the relationships between volumetric measures of hypothalamic subunits and plasma hormonal metrics (LH, FSH, T, and P4) across biological sex and cognitive status groups with SPSS v28.0.11. Independent variables included volumetric measures of specific hypothalamic subunits, while dependent variables were the plasma levels of the hormones LH, FSH, T, and P4. Grouping variables included biological sex (male, female) and cognitive status (CN, MCI, AD). The regression models were checked for assumptions of normality, linearity, and homoscedasticity. Statistical significance was determined using α = 0.05. The coefficient of determination (R^2^) was reported for each model to quantify how well the independent variables explained the variance in the dependent variables.

## 3. Results

### 3.1. Demographic and Clinical Information

Participants (*n* = 493) were categorized into three diagnostic groups: cognitively normal (CN, *n* = 54), mild cognitive impairment (MCI, *n* = 343), and Alzheimer’s disease (AD, *n* = 96). The mean age across groups was approximately 75 years (See Table 1). The biological distribution varied between groups, with the CN group comprising ~50% females, the MCI group 35% females, and the AD group with 45% males. On average, participants had completed 15.5 years of formal education. A trend was observed CDR-SB scores, with a significant increase from the CN group to the MCI group, peaking in the AD group. MMSE scores exhibited a decline from CN to MCI to AD, indicating deteriorating cognitive function. A similar downward trend was seen in RAVLT scores across the groups. The estimated total intracranial volume remained relatively consistent between groups.

### 3.2. Volumetric Findings

**Figure 1:**
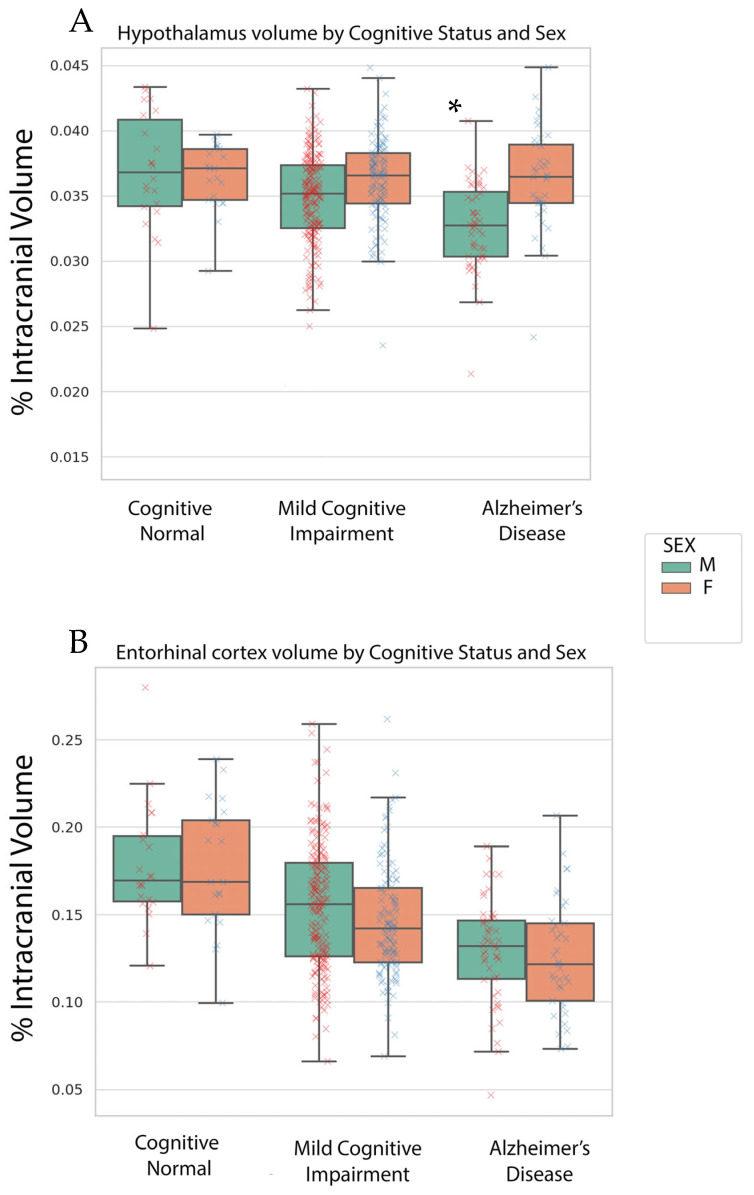
Box plots of hypothalamic (**A**) and entorhinal volume (**B**) across cognitive status on the x-axis. Green boxes are male plots and orange boxes are female plots. Numerical values in box plots are the % of intracranial volume reflective of the unique condition. A red x indicates a male (M) point and a blue x indicates an individual female (F) point. The asterisk indicates the significance at *p* < 0.05 of the sex-by-cognitive status interaction.

Our multivariate ANCOVA revealed a cognitive status effect on entorhinal, fusiform, hippocampal and hypothalamus volume (*p*’s < 0.01). The volume decreased with worsening cognitive status. A biological sex-by-cognitive status was only found for hypothalamus volume (*p* < 0.01, See Figure 1). The hypothalamus volume stayed relatively the same in women across cognitive status; however, hypothalamic volume decreased with worsening cognitive status for men. The hypothalamic differences between biological sex groups were in the MCI and AD groups.

### 3.3. Plasma Hormone Regression Analyses Findings

Our multiple linear regression analyses revealed distinct subunit volumes were associated with hormone plasma levels of FSH, LH, P4, and T across biological sex groups (see Table 2 and Table 3). For females within CN & MCI groups, posterior hypothalamus, anterior–superior hypothalamus and tuberal regions were found to be significantly associated with FSH, LH, P4, and T (See Table 2) and inferior tuberal hypothalamus regions were associated with FSH and LH hormone levels in AD females. Table 2 outlines the significant predictors among hypothalamic sub-units for different stages of cognitive status in females, specifically focusing on their relationship with levels of various hormones: FSH, LH, P4, and T. Each hormone is associated with specific hypothalamic sub-units that serve as significant predictors (with *p* < 0.05) for changes in cognitive status, as determined through multiple regression analyses. The squared multiple correlation coefficient (R^2^) is provided for each significant predictor, indicating the proportion of variance in cognitive status that can be explained through the respective hypothalamic subunit’s activity in relation to the hormone level. It appears cognitive status worsens the relation of the hypothalamic units and circulating hormonal status. Inferior tuberal regions have a moderate impact on the variance of progesterone (38%) and testosterone (20%) hormonal levels in cognitive normal females. The posterior hypothalamus was also revealed to have a moderate impact on the variance of LH (21%) hormonal levels. In AD females, inferior tuberal regions have a small yet significant impact account for 11% and 12% of the variance in FSH and LH levels.

For males, the posterior hypothalamus and tuberal regions were found to be significantly associated with FSH, LH, P4, and T across just CN and MCI groups, where the AD process seems to diminish the effect of hypothalamic subunits’ associations of hormonal plasma levels, with no hypothalamic units predicting levels in AD males (see Table 3). Table 3 contains similar representative information as Table 2. For males, the posterior hypothalamus had a moderate association with LH, accounting for 21% of the variance. The inferior tuberal hypothalamus had a moderate association with P4, accounting for 18% of the variance and the superior tuberal hypothalamus had a distinct moderate impact on T plasma hormone levels. The association decreased in MCI males, with the posterior hypothalamus region having a lower association (6%) with LH plasma levels, while the inferior tuberal hypothalamus with testosterone accounted for 5% of the variance and the combination of the inferior tuberal and posterior hypothalamus accounted for 6% of the variance in FH plasma levels.

## 4. Discussion

Consistent with previous literature, we observed a significant effect of cognitive status on hippocampal and entorhinal cortex volumes [18,19]. Furthermore, our data revealed a biological sex-by-cognitive status interaction for hypothalamic volume, with reductions observed across cognitive groups in males but not in females. In males within CN and MCI groups, volumes of the posterior hypothalamus significantly predicted gonadotropin levels, while the inferior tuberal region was predictive of sex hormone levels. Conversely, in females, different hypothalamic subregions were associated with circulating sex hormones across different cognitive statuses [20]. It also appeared that the strength of the association of hypothalamic subregions with plasma hormone levels decreased with worsening cognitive status groups, especially in males with no significant hypothalamic subunit predictors in the AD males.

Recent investigations have unveiled associations between volumetric changes in specific hypothalamic subregions and alterations in hormone levels, with notable distinctions across cognitive states and biological sex [21,22]. In CN males, increased volumetric values within the superior tuberal regions, comprising the dorsomedial nucleus, paraventricular nucleus, and lateral hypothalamus, have been positively correlated with increased T levels. This relation underscores the pivotal role of these regions in neuroendocrine homeostasis and their potential as biomarkers for cognitive health [23]. Conversely, males within MCI groups exhibit an inverse relationship between the volume of inferior tuberal regions, including the infundibular nucleus and ventromedial nucleus, and T levels, implicating these sites in the neuroendocrine dysregulation associated with cognitive decline [23,24]. This is also demonstrated with the decrease in the strength of association with worsening cognitive status with no predictors found in AD males. In cognitively normal individuals, optimal testosterone levels may enhance the posterior hypothalamus region’s functionality, supporting alertness and cognitive processing efficiency. However, in conditions like MCI and AD, alterations in testosterone levels could disrupt these processes, contributing to cognitive symptoms such as disorientation, memory impairment, and disrupted sleep-wake cycles.

Males also exhibited enlarged posterior hypothalamic volumes, encompassing the mamillary bodies and lateral hypothalamic areas, correlating with decreased LH levels in both CN and MCI subjects. This phenomenon suggests a putative compensatory adjustment or pathological decline in hormonal regulation associated with changes in regions traditionally implicated in memory and arousal functions [25,26].

Paralleling these findings, females across various cognitive status groups have exhibited distinct neuroendocrine profiles. In CN females, increased inferior tuberal volumes are associated with elevated P4 and T levels, revealing a moderate association ranging from approximately 20 to 40% of the variance, reinforcing the notion of a sex-specific regulatory mechanism operative in cognitively intact females [27]. However, in the context of AD, a similar increase in inferior tuberal volume correlates with increased LH and FSH levels with approximately 5% of the variance, potentially reflecting an adaptive neuroendocrine response to progressive neurodegeneration [28].

Further, multifaceted effects emerge when evaluating the posterior hypothalamic volume in females with CN, where reductions are paradoxically linked with increased LH, indicating potential feedback dysregulation [29]. In biological sex females with MCI, the landscape shifts; expansions in anterior superior volumes, which include the preoptic area and PVN, are related to FSH decrements, whereas increments in superior tuberal volume align with reduced P4 levels, hinting at a disrupted neuroendocrine homeostasis as cognitive impairment advances.

Our findings also suggest that distinct units may interact with circulation plasma hormones to promote cognitive function in males and females. Specifically, the differential associations between various hypothalamic subregions and hormone levels across groups point to a complex, sex-specific orchestration of neuroendocrine regulation that impacts cognitive abilities. For example, in females, the significant correlation between the anterior, superior and inferior tuberal regions with FSH and LH, respectively, across different cognitive statuses, underscores the role these hormones and hypothalamic regions may play in cognitive maintenance and decline. Similarly, in males, the involvement of the posterior hypothalamus and inferior tuberal regions in relation to LH and FSH levels, respectively, across cognitive states, suggests a tailored neuroendocrine interaction that supports cognitive functions. These observations imply that targeted modulation of specific hypothalamic-hormonal pathways may offer new avenues for cognitive enhancement and neuroprotection, with potential implications for personalized interventions in neurodegenerative diseases and cognitive impairments.

Collectively, these findings propel our understanding of the hypothalamic roles in neuroendocrine function across cognitive statuses in AD continuum. There are moderate associations among distinct hypothalamic subregions, mostly the posterior hypothalamus and tuberal hypothalamus regions, and distinct sex and gonadotropic hormones in the cognitive normal group across males and females. These associations, although significant, decreased in MCI and AD with males showing no association with the hypothalamus subregions and any of the hormones utilized in this study. Interestingly, hypothalamus volume in AD was able to distinguish differences between males and females. Future work, necessitating longitudinal analyses, functional imaging, and mechanistic explorations, promises to elucidate the complexities of these relationships. This may set the groundwork for future precision related work in AD care and treatment.

## Figures and Tables

**Table 1:** Demographic and clinical information across cognitive status.

Group	N	Age (Years)	Sex (% Female)	Education (Years)	CDR-SB	MMSE	RAVLT	eTIV (Liters)
Cognitive Normal	54	75.3 ± 5.9	28/26 (48%)	15.6 ± 3	0.1 ± 0.3	29.1 ± 1.2	35.6 ± 34.9	1.51 ± 0.15
Mild Cognitive Impairment	343	74.9 ± 7.3	223/120 (35%)	15.7 ± 3	2.2 ± 1.4	26.4 ± 2.9	67.6 ± 31.4	1.58 ± 0.15
Alzheimer’s Disease	96	75.1 ± 7.9	53/43 (45%)	15.2 ± 3	5.6 ± 2.7	21.3 ± 4.6	85.2 ± 25.6	1.57 ± 0.20
Totals & Statistics	-	F = 0.09, *p* = 0.92	χ^2^ = 5.5, *p* = 0.06	F = 1.2, *p* = 0.30	F = 223.1, *p* < 0.001	F = 132.5, *p* < 0.001	F = 44.6, *p* < 0.001	F = 3.0, *p* = 0.049

CDR-SB = Clinical Dementia Rating-Sum of Boxes, eTIV = estimated intracranial volume, MMSE = Mini Mental State Exam, SD = Standard Deviation. Age values are mean.

**Table 2:** Significant hypothalamic sub-unit predictors (*p* < *0*.05) from multiple regression analyses across cognitive status for females.

	Cognitive Normal	MCI	AD
Follicle Stimulating Hormone	N/A	Anterior-superior (R^2^ = 0.05)	Inferior Tuberal (R^2^ = 0.11)
Luteinizing Hormone	Posterior Hypothalamus (R^2^ = 0.21)	N/A	Inferior Tuberal (R^2^ = 0.12)
Progesterone	Inferior Tuberal (R^2^ = 0.38)	Superior Tuberal (R^2^ = 0.08)	N/A
Testosterone	Inferior Tuberal (R^2^ = 0.20)	N/A	N/A

AD = Alzheimer’s disease, MCI = mild cognitive impairment, N/A= not available, R^2^ = coefficient of determination.

**Table 3:** Significant hypothalamic sub-unit predictors across cognitive status for males.

	CN	MCI	AD
Follicle Stimulating Hormone	N/A	Inferior Tuberal & Posterior Hypothalamus (R^2^ = 0.06)	N/A
Luteinizing Hormone	Posterior Hypothalamus (R^2^ = 0.21)	Posterior Hypothalamus (R^2^ = 0.06)	N/A
Progesterone	Inferior Tuberal (R^2^ = 0.18)	N/A	N/A
Testosterone	Superior Tuberal (R^2^ = 0.21)	Inferior Tuberal (R^2^ = 0.05)	N/A

R^2^ = coefficient of determination, N/A = not available, CN = cognitive normal, MCI = mild cognitive impairment, AD = Alzheimer’s disease.

## Data Availability

Data used in preparation of this article were obtained from the Alzheimer’s Disease Neuroimaging Initiative (ADNI) database (adni.loni.usc.edu). As such, the investigators within the ADNI contributed to the design and implementation of ADNI and/or provided data but did not participate in analysis or writing of this report. A complete listing of ADNI. investigators can be found at: http://adni.loni.usc.edu/wp-content/uploads/how_to_apply/ADNI_Acknowledgement_List.pdf (accessed on 15 May 2023).
